# Stakeholders’ Perspectives on Quality Measurement of Oral Health Care in the Netherlands: A Qualitative Study

**DOI:** 10.1016/j.identj.2025.03.004

**Published:** 2025-04-01

**Authors:** Amy Righolt, Denise Duijster, Kirsten Smits, Anke Oerlemans, Philip van der Wees, Stefan Listl

**Affiliations:** a Department of Dentistry – Quality and Safety of Oral Healthcare, Radboud University – Radboudumc (RIHS), Nijmegen, The Netherlands; b Capaciteitsorgaan (Council for Medical Manpower Planning), Utrecht, The Netherlands; c Department of Oral Public Health, Academic Center for Dentistry Amsterdam, University of Amsterdam and VU University, Amsterdam, The Netherlands; d Knowledge Institute of the Dutch Association of Medical Specialists, Utrecht, The Netherlands; e IQ Health and Department of Rehabilitation, Radboud University Medical Center, Nijmegen, The Netherlands; f Section for Oral Health, Heidelberg Institute of Global Health, Heidelberg University Hospital, Heidelberg, Germany

**Keywords:** Oral health, Quality of care, Quality measures, Dental professionals, Qualitative research

## Abstract

**Objective:**

This study aimed to identify which barriers and facilitators exist and can be expected when measuring quality of oral health care according to different stakeholders in the Netherlands.

**Methods:**

A total of 36 semistructured interviews were conducted with dentists, patients, universities and knowledge institutes, health insurance companies, professional dental associations, and governmental health organisations. Using qualitative content analysis, barriers and facilitators were classified according to the frameworks of Grol and Cabana.

**Results:**

In total 70 barrier and 53 facilitating factors were identified in the 5 domains of the frameworks. Various stakeholders found quality measurement challenging because the quality of oral health care is difficult to define with a lack of consensus on what constitutes quality of oral health care. Patients mentioned that, for them, quality of oral health care is difficult to assess. Dentists experienced a fear of being monitored and were apprehensive of the administrative burden of quality measurement. On an organisational level, the isolation of dentistry from the medical field was mentioned as a barrier. Facilitating factors were discussing quality in a trusted environment, and developing more clinical practice guidelines, which include meaningful quality measures.

**Discussion:**

This study identified barriers and facilitators for measuring quality of oral health care in the Netherlands. Findings signal the importance of achieving consensus on the definition of quality of oral health care. Further strategy discussions about how quality of oral health care can be made insightful in a way acceptable to all stakeholders are needed to make progressions in quality improvement.

## Introduction

Oral diseases remain a major public health burden worldwide and the economic impact of oral diseases is substantial.^1^, ^2^, ^3^, ^4^ To maximise the quality of oral health care given the fact that resources are scarce, it is pivotal to provide routine feedback information on the quality of oral health care. Feedback information can help patients, health care providers, and policy makers make well-informed choices to improve oral health care quality. A frequently used method to provide feedback information is by the use of quality measures. A quality measure is a “measurable element of practice performance for which there is evidence or consensus that it can change the quality of health care provided”.^5^ These measures can be used to provide feedback data on the structures, processes, and outcomes of oral health care. However, thus far, data from oral health care quality measures are not routinely collected in the Netherlands.

The availability of data from reliable and meaningful measures can contribute to a more transparent health care system and help guide the process of decision making about quality improvement in oral health care. Routinely collected data can be valuable for patients to determine which care aligns best with their needs and may help patients monitor their health and health care pathways.^6^^,^^7^ For dental professionals and policy makers, comparative feedback data has the potential to illustrate where improvements are possible and whether the provided care is consistent with current professional knowledge.^6^

In recent years, more attention has been devoted to quality measurement and the development of quality measures. In many parts of the medical field, quality measures are now institutionalised and part of a standard practice within and across health care systems.^8^ Although societal expectations towards the quality of oral health care continue to increase and many quality measures have been developed, the routine use of quality measures in oral health care is not widespread.^6^^,^^9^^,^^10^

There may be several factors that have been obstructing the implementation of quality measures in oral health care, for example, barriers related to measurement, data privacy, logistics, and stakeholder perceptions and concerns. Understanding these barriers is essential for making progress towards quality improvement. It is important that perceptions of different stakeholders are explored, such as patients, dentists, insurers, and dental institutes, as each group has unique insights and concerns that must be considered to create a feasible and acceptable approach to quality measurement. Therefore, the aim of this study was to identify which barriers and facilitators exist and are to be expected with regard to measuring quality of oral health care according to different stakeholders in the Netherlands. By identifying and addressing the barriers and facilitators, tailored implementation strategies can be developed that facilitate the adoption of quality measures, leading to a more transparent and effective oral health care system.

## Methods

### Setting and study design

In this study, the barriers and facilitators of oral health care quality measurement in the Netherlands were qualitatively explored. Semistructured interviews were conducted with individuals from 6 stakeholder groups within the Dutch oral health field: (1) dentists, (2) patients, (3) universities and knowledge institutes, (4) health insurance companies, (5) professional dental associations, and (6) governmental health organisations and departments. The interviews were conducted between September 2019 and June 2020. For this study, a waiver was obtained from the Medical Ethical Committee of the Radboud University Medical Center Nijmegen (file number 2019-5708).The Consolidated criteria for reporting qualitative research (COREQ) were used as a standard for reporting.^11^

### Participants and recruitment

#### Dentists and patients

Purposive sampling was used to select the dentists participating in the study. The goal was to include a group of dentists from the Netherlands with varying characteristics with regard to age, gender, education, location, and practice region. Dentists were recruited by telephone or e-mail via the author's network and snowballing. Prior to the study a professional work-related relationship existed between 2 of the dentists and the interviewer. One of the invited dentists refused to participate due to time constraints. Participating patients were recruited by mail or e-mail via dentists and via a patient panel of the Radboud University Medical Center. There was no personal connection between the researchers and patients. In addition, a patient representative was invited. Similar to the dentists, purposive sampling was used with respect to age, gender, and region of the patients. For both groups, the number of persons interviewed was not defined in advance but was dependent on data saturation, meaning, the point at which no new information was obtained from the interviews.^12^

#### Other stakeholders

Representatives from 4 other stakeholder groups were invited to participate: the 4 largest health insurance companies in the Netherlands, all 3 dental schools in the Netherlands, 1 knowledge institute for oral health, the 2 professional dental associations, and 4 governmental health organisations and departments. The representatives approached were all working on quality of oral health care within their function.

### Data collection

Prior to the interviews, all participants received a letter with information about the research aim and procedure. Written and verbal consent for participation was obtained. At the start of each of the interviews, it was mentioned that the interviews could be stopped at any moment without giving a reason. The developed interview guide was discussed with all authors and pilot tested with a convenience sample of 2 patients and 2 dentists. The interview guide for each participant group can be found in Appendix A. Each interviewee received questions about quality of oral health care that focused on 4 topics: (1) perceptions of the meaning of quality of oral health care, (2) measuring quality of oral health care, (3) domains of quality of oral health care, and (4) the ideal way to measure quality of oral health care. Examples of questions were “According to you, what constitutes quality of oral health care?,” “What makes it difficult to measure quality of oral health care?,” and “What could facilitate oral health care quality measurement?”. The domains of quality of oral health care were structured on the basis of the framework of the Institute of Medicine (IOM) and a previous study in which a working definition for quality of oral health care was defined.^9^^,^^13^ The interviews with patients were held via telephone. The interviews with other stakeholders and dentists were held face-to-face at a location of their preference, either at the workplace of the stakeholder, the workplace of the first author or house of the participating dentist. First the authors AR, DD, and KS conducted 2 interviews per researcher. The remaining interviews were conducted by the first author (AR). No repeat interviews were carried out. The interviewers aimed for rich descriptions. The transcripts were not returned to participants for comments or correction but were available on request. Only the primary researchers had access to the data.

### Data analysis and rigor

The interviews were audiotaped, transcribed verbatim and qualitatively analysed with ATLAS.ti (version 22.0.11). Fieldnotes were made after the interviews. The transcripts of the interviews were analysed using qualitative content analysis and took into account both the direct meaning and the underlying meaning of the text. To enhance thoroughness and prevent the risk of potential blind spots, 2 researchers (AR and DD) read, re-read and coded the first interview of every stakeholder group independently. The codes were compared, and discrepancies were discussed until consensus was reached. Themes and codes were derived from the data. The interviewees did not provide feedback on the findings. Peer debriefing took place on a biweekly basis (AR/DD/KS). In peer debriefing discussions, the main findings, minor themes, and diverse cases were discussed. The codes were categorised according to 2 theoretical frameworks, the frameworks of Grol^14^ and Cabana.^15^ Both frameworks facilitate the categorisation of barriers and incentives for change in evidence-based clinical practice, which may help to develop effective implementation strategies. The frameworks were combined according to previous research^16^^,^^17^ and the combined frameworks facilitated the classification of potential barriers and facilitators into 5 domains: (1) factors related to the guideline/quality measurement (2) factors related to the dental professional, (3) factors related to the patient, (4) factors related to the organisation/system and (5) social setting factors (eg, colleagues of the dental professionals). The final barriers and facilitators found were discussed in meetings with multiple authors (DD, KS, SL, PW, AR).

### Trustworthiness

The 4 components of trustworthy qualitative research were incorporated in the design: credibility, dependability, confirmability, and transferability.^18^ Extensive use of quotes from interviewees contributed to the authenticity and credibility of the description of the barriers and facilitators, and illustrate the consistency between data and the findings. Researcher triangulation was used during the data collection and analysis. Independent open coding and re-coding by 2 authors (AR/DD) was used, group discussions were held, and all researchers provided feedback on the writing process to enhance credibility and dependability. Transferability of the data was improved by the provision of context information and thick description. Diversity in the backgrounds of the research team contributed to the confirmability of the study. The first author AR (female, PhD candidate, dental hygienist, and dental public health researcher) was qualitatively trained. The second analysing researcher DD is a dental public health researcher and had previous experience with qualitative research (female, PHD, associate professor). The other researchers had a background in public health (KS, female, PhD, postdoc), dentistry and health economics (SL, male, PhD, professor), physical therapy (PW, male, PhD, professor) and ethics (AO, female, PhD, assistant professor). PW and AO were also qualitatively trained.

## Results

### Participants

In total 36 interviews were conducted. The face-to-face interviews lasted 30 to 75 minutes, the telephone interviews with patients lasted 20 to 40 minutes. Eleven dentists, ten patients from across the Netherlands and a patient representative were interviewed. For these 2 stakeholder groups, no new barrier and facilitating topics occurred in the final 2 interviews and saturation was reached. Table 1 shows the characteristics of the participating dentists and patients. Furthermore, 14 other stakeholders across 4 stakeholder groups participated (universities and knowledge institutes, health insurance companies, professional dental associations, and governmental health organisations and departments. For these groups it was not possible to reach data saturation, since there were no similar stakeholders to be interviewed. New data from these interviews still emerged until the final interview.

**Table 1:** Characteristics of participating patients and dentists.

Characteristics of participating patients*		
*Variable*		(n = 10)
Age, y	Mean (range)	54.7 (29-75)
Gender, n	Women	6
	Men	4
**Characteristics of participating dentists**		
*Variable*		(n=11)
Age, years	Mean (range)	39.6 (27-65)
Gender	Women	5
	Men	6
Years active	Mean (range in years)	19.6 (2-38)
Type of practice	Solo practice	3
	Group practice	4
	Corporate dental chain	3
Education location	ACTA, Amsterdam	2
	Radboud University, Nijmegen	3
	Groningen, University of Groningen	4
	Utrecht, University of Utrecht	1
	Foreign universities	1
Practice region	North of the Netherlands	4
	Middle of the Netherlands	4
	South of the Netherlands	3

⁎ Excluding a patient representative, who was a representative of patients

### Barriers and facilitators

Barrier and facilitating factors were grouped into overarching topics and later categorised into the 5 domains of the theoretical frameworks. In total 70 barrier and 53 facilitating factors were identified in the 5 domains. Most barriers were found for ‘topics and factors related to the organisation/system’ and ‘topics and factors related to the dental professional’. Overall, frequently mentioned barrier topics were *acceptance, definition, fear, data,* and *feedback*. Frequently mentioned facilitating topics were related to *implementation, IT infrastructure, guidelines,* and *data*. Table 2 provides an overview of all barriers and facilitators related to quality improvement in oral health care according to all stakeholders. All topics and factors were classified based on the 5 domains of the 2 theoretical frameworks. Appendix Tables 1 to 6 provide an overview of the barriers of each of the stakeholder groups separately. Figure presents quotes from all stakeholders for each of the 5 predetermined domains.

**Table 2:** Barriers and facilitators related to quality improvement in oral health care according to different stakeholders

Domain / *topic*	Barrier factors	Facilitating factors
Topics and factors related to measurement and guidelines		
*Data*	Lack of reliable data	The presence of data about objective observations
	Expectations that feedback data is not useful	Comparable patient populations
	Lack of data availability	The availability of (feedback) data
		Up-to-date patient records
*Definition*	Quality is perceived as subjective	
	Other factors have an influence on quality of oral health care	
	Quality is not measurable	
	Unknown influence of dentist on the health outcome of the patient	
*Patient satisfaction*	Lack of usable patient satisfaction data	
*Implementation*		No value judgement or sanctions when measuring quality will increase willingness to participate in quality improvement initiatives
		Accessible quality improvement information and initiatives
*Education*		More education about quality measurement in dental curricula
*Guidelines*	Lack of quality and availability of clinical practice guidelines	Availability of clinical practice guidelines
	Wide range of professional standards in clinical practice guidelines	Clear care options in clinical practice guidelines to facilitate evidence informed decision making
	Lack of a clear goal	Availability of a clear goal and care plan
	Guidelines change quickly over time	
*Quality indicators*	Absence/incorrect quality indicators	
	Limited evidence base	
Topics and factors related to the dental professional		
*Feedback*	Dental professionals not open to feedback	Mindset shift to being open to feedback
	Feedback believed to be unnecessary	
	Feedback data difficult to interpret	
	Absence of reflective skills of dentists	
	Overestimating one's own abilities	
*Implementation*	Participation obligation	Voluntary participation
	Professional autonomy regarding willingness to improve quality of care	
*IT infrastructure*		Non-labor intensive
*Quality improvement*	Absence of intrinsic motivation of dentists	
*Patient centeredness*	Strong professional autonomy hinders communication with patients and willingness to improve quality	
*Patient satisfaction*	Information about patient satisfaction perceived as unnecessary	
*Regulation*	Fear of regulations	
	Knowledge gap about regulations	
*Acceptance*	Resistance of the occupational group	
*Fear*	Fear of being monitored	
	Fear of quality indicators being used for enforcement	
	Fear of quality indicators being used as a sanction	
*Workflow*	Administrative burden	
	Additional workload	
	Additional time investment	
*Variation*	Lack of comparability of results/practices	
*Change*	Lack of adaptation to the times	
	Lack of acceptance of change	
	Lack of feeling responsible for one's own work due to many changes in personnel	
*Continuing education*		Participation in (quality of oral health care related) continuing education
Topics and factors related to the patient		
*Patient centeredness*	Lack of transparency of treatment plans and costs	Improve transparency of treatment plans and costs
		Facilitate online comparison of experiences with the provided care
*Patient satisfaction*	Survey fatigue	Provide simple options for patients to give feedback
	Quality difficult to assess for patients	Useful questions
	Lack of anonymity experienced by patients	Find ways to reach younger people in patient satisfaction surveys
*Guidelines*		Availability of patient information
Topics and factors related to the organisation/system		
*Data*	Fear data ends up in the wrong hands	Comparable on a large scale
	General data protection regulation makes it harder to gather data about quality of care	Recording use of the data
		Clear data ownership
*Definition*	Difficult to define quality of oral health care	Availability of a definition
	No consensus regarding the definition	
	Lack of a general vision of what constitutes high quality care	
	Conflicting interests	
*Feedback*	Pedantic/didactic	Positive feedback
*Finance*	Costs of measuring quality	Detach quality and finance
	Current financing system does not promote measuring quality	
	Link quality to finance	
*Implementation*	Dentistry is isolated	Enthuse quality improvement among all stakeholders
	Influence of professional dental associations	Acknowledgement /rewarding quality improvement activities
	Financing of quality improvement	National or European implementation
		Participation obligation
*IT infrastructure*	Absence of IT infrastructure facilitating data collection for feedback information	Presence of IT infrastructure to facilitate data collection for feedback
	Possibility central monitoring	Automated systems
		Checklist in electronic health records
		Electronic health record filing
		Link between systems
		Quality components in electronic health record software
*Quality improvement*	Absence of quality thinking among dentists	Reward quality
		Warranty on delivered care
		Independent quality institute
		Availability of benchmark information Feedback data for comparisons with peers
		Improvement cycles
		Quality vison about what needs to be done to deliver high quality care
*Education*	Lack of attention for quality in the curriculum	Encourage quality thinking
		One dental curriculum in the Netherlands
		Independent policy/no conflict of interest
*Patient satisfaction*		Digitalisation
*Regulation*	Frequent changes	Provision of clear information about regulations
	Information overload	
	Sole focus on hygiene as indicator for quality	
*Acceptance*	Distrust in health insurance companies and governmental health organisations/departments	
	Lack of support	
	Resistance of professional associations	
	Lack of consensus on what constitutes good oral health care	
*Quality indicators*	Distorted picture because of use by health insurance companies	
*Variation*	Variation in patient characteristics	
*Change*	Frequent changes of boards and visions	
Topics and factors related to the social setting		
*Feedback*		From colleagues
*Implementation*		Start small
		Discuss results in a trusted environment
		Compare results with colleagues
*Quality improvement*		Address willingness to discuss quality improvement
Education		Encourage attention for teamwork and reflection
Communication		Facilitate discussions about quality of oral health care

**Figure:**
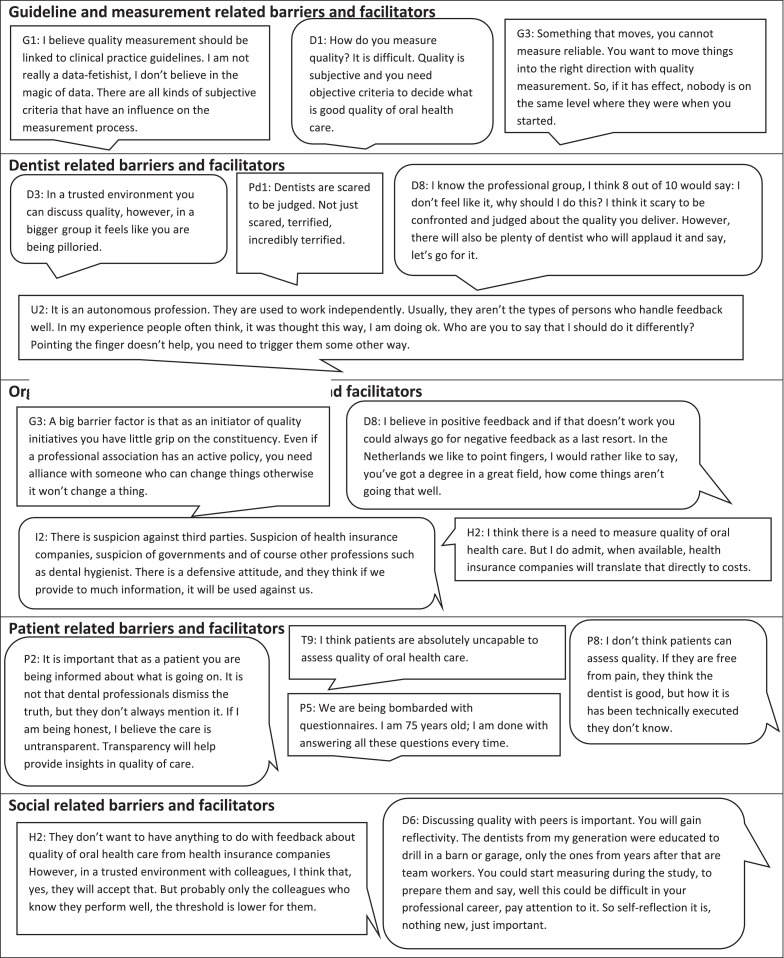
Illustrative quotes from various stakeholders concerning barriers and facilitators of measuring quality of oral health care.

*1. Barriers and facilitators related to quality measurements and guidelines*

The guideline and quality measurement barriers mentioned by the interviewees were related to the topics of *data, definition, quality indicators,* and *guidelines*. Regarding *data*, barriers mentioned were a lack of reliable data, the data was not experienced as useful, or a lack of data availability was perceived. With regards to a *definition* of quality of oral health care, especially dentists and patients mentioned that quality of oral health care is difficult to define. For example, one of the dentists said:

*Dentist 2: ‘’I really don't know how to measure quality. Quantity is no problem of course, but quality is very difficult to measure”.*

Other stakeholders frequently mentioned that the difficulty of measuring quality is due to a lack of consensus on quality of oral health care and conflicting interest of involved stakeholders in quality improvement initiatives. In addition, interviewees often mentioned that quality of oral health care is subjective or not measurable (see Figure). It was frequently mentioned that clinical practice guidelines can foster quality improvement through the provision of professional standards. Therefore, it was suggested that quality measurement should be linked to clinical practice guidelines (see Figure). Most of the interviewees pointed out that the availability and quality of clinical practice guidelines in oral health care is often limited and they emphasised the necessity to frequently update the clinical practice guidelines to the current professional standards. Some mentioned that clinical practice guidelines frequently lack a clear goal. For quality indicators interviewees, except for patients, experienced an absence of acceptable and valid quality indicators with a substantial evidence base.

Facilitators in the guideline and quality measurement domain were classified as related to the topics of *data, implementation, education,* and *guidelines*. To facilitate quality measurement, the majority of the interviewees mentioned that there is a great need for the availability of data. For the data collection, interviewees emphasised the need for objective observations and up-to-date patient records. Some mentioned the necessity of benchmark data about outcomes of care for practices with similar patient populations. Many interviewees reported that the implementation of quality measurement could probably count on more support if they were implemented without judgment or sanctions:

*Governmental health organization 4: “Being judged on quality would be killing, we do not have enough insights in quality of care. If you use sanctions, you will not get quality, people are not going to be transparent.”*

Other facilitating factors mentioned were more educational about quality measurement in dental curricula, which could enhance a focus on quality improvement later in life, and the development of more high-quality clinical practice guidelines with measurable elements of care.

*2. Barriers and facilitators related to the dental professional*

Barrier topics mentioned related to the dental professional were feedback, implementation, quality improvement, patient-centeredness, patient satisfaction, regulation, acceptance, fear, workflow, variation, and change. Most interviewees, both dental professionals and other stakeholders, noted that they thought the professional group would not be open to feedback due to the fact they think it is either unnecessary, have limited reflective skills, or they overestimate one's own abilities. The majority of interviewees mentioned that there is a fear of being monitored or they assume that quality measurement comes with a large administrative burden or additional workload (see Figure). It was also pointed out by younger dentists and other stakeholders that older dentists have difficulty accepting change:

*Dentist 4: “I think we need to wait until the current older dentists are retired, we need to start with quality improvement early in the dental curricula so that people know it is part of the job. And yes, this is not something you can arrange in the next two years.”*

Further, some mentioned that the fact that it can be an autonomous profession could hinder the willingness to participate in quality improvement initiatives (see Figure). Facilitators mentioned were mainly related to the IT infrastructure, implementation, and a needed culture change with regard to being susceptible to feedback. Frequently it was mentioned that dental professionals were more likely to participate in quality improvement initiatives if their involvement was non-labor intensive due to a well-functioning IT infrastructure. It was also noted that voluntary participation, preferably in a trusted setting, could contribute to a wider support for quality measurement.

*3. Barriers and facilitators related to the patient*

The barriers and facilitators in the domain of patient-related topics and factors were mostly mentioned by patients themselves. The ones related to the topic of patient satisfaction focused on survey fatigue and how difficult it is for patients to assess quality (see Figure). Some patients mentioned they felt that concerns regarding anonymity were a large barrier to provide feedback about the quality of oral health care:

*Patient 7: “I feel that when you provide negative feedback after a treatment, the dentist will probably know who the feedback is from, it does not feel anonymous.”*

A lack of transparency about the costs of care and about the execution of the provided care was frequently mentioned as a barrier. Facilitating factors mentioned were the improvement of transparency, the facilitation of online comparisons of experiences with provided care, and measuring patient satisfaction using simple and useful questions. Further it was stressed by patients that there is a need for information written specifically for patients in clinical practice guidelines.

*4. Barriers and facilitators related to the organisation/system*

Most barriers and facilitators were found for the domain organisation and system-related topics and factors. One main impeding implementation barrier pointed out was that dentistry is isolated from the rest of the medical field and in some cases also from other dentists:

*Health insurance company 4:* “*Dentists do not like to let other people take a look behind their curtain. They are individualists, at least for the most part. They think; I am in my own practice minding my own business and health insurance companies, the health care inspectorate, and government should not interfere.”*

Other noted barrier factors related to the success of implementation are the financing of quality improvement and the strong influence of professional dental associations in the Netherlands. For example, one of the governmental health organisations mentioned:

Governmental health organisation 2: “It is difficult to get consensus on outcome measures if you leave it up to dental *professional associations. It is crucial to detach content related decisions from material interest groups. History proves that if you leave it up to the professional dental associations that is not possible.”*

Other organisation and system-related factors that concerned many of the interviewees were the lack of a definition and an IT infrastructure that facilitates comparison between practices and quality improvement. Acceptance emerged as a topic due to a distrust in health insurance companies and governmental health organisations and departments, a lack of consensus on the quality of oral health care, a lack of support from various stakeholders, and more specifically several interviewees mentioned a resistance from professional dental associations. Dentists frequently mentioned that there are too many changes in regulations and that they experience an information overload. Concerning the financing system, it was both mentioned as a facilitator and a barrier to detach or link quality and the financing system of oral health care. In general, interviewees were not satisfied with the current financing system of oral health care, but few alternatives were suggested. Also, opinions differed with regard to whether or not oral health care should be covered in the basic insurance coverage.

The main facilitators related to organisations and/or the system concern the topics of implementation, quality improvement, data, and IT infrastructure. Many dentists mentioned that a great facilitator would be to enthuse quality improvement and to acknowledge or reward quality improvement. For example, dentist 8 noted:

*Dentist 8: “One way to stimulate quality improvement is to reward dentists who invest in quality improvement and perform well. This could be a financial reward, but you could also think about other ways of rewarding quality care.”*

Dentists noted that an obligation to participate in quality improvement initiatives would result in a counterproductive effect. Quality improvement initiatives should start small and focus on the intrinsic motivation of dental professionals. Among the governmental health organisations and health insurance companies, it was frequently mentioned that implementation of quality improvement initiatives would benefit from obligatory participation and should focus on how to enthuse the dental field for quality improvement. With regard to the IT infrastructure, almost every interviewee mentioned that quality measurement should be made as simple as possible for participating dental professionals. Factors mentioned were automatic data collection, the presence of a working IT infrastructure, a link between different electronic health record systems (EHRs) or checklists in EHRs.

*5. Barriers and facilitators related to the social setting*

For the social setting factors, no barrier topics emerged. Facilitating factors were to make the topic of quality open for discussion. Dentists and other stakeholders pointed out to discuss quality in a trusted environment, and that quality measurement can count on the most support when results are compared and discussed with colleagues (see Figure). For the future, many interviewees mentioned it would be beneficial if dental schools focus more on teamwork, reflection and quality thinking in the dental curricula:

*Dentist 6: The reflectivity of dentists is mediocre. The average dentist my age was taught to drill in a barn or garage, since a few years there are dentists who focus on teamwork. I think if there would be more attention for reflection and feedback in the dental curriculum later in their professional life they will be more open to feedback from colleagues.”*

## Discussion

This is the first study to describe an in-depth analysis of which barriers and facilitators exist for measuring quality of oral health care according to various stakeholders in the Netherlands. This study confirms that a lot of obstacles are to overcome before quality measurement in the Dutch dental field can be practically executed and is to be accepted by the involved stakeholders. The findings of this study confirm that quality measurement is challenging because of a lack of a definition and consensus on what constitutes quality of oral health care. From the perspective of the dentists, impeding factors of quality measurement were a fear of being monitored and a fear of an additional administrative burden. For patients an important barrier exists in the difficulty to assess quality. In addition, the necessity of more information regarding the provided care and the costs of care were emphasised by patients. On an organisational level it was shown that dentistry is frequently isolated from the rest of the medical field and sometimes also from other dentists. It became apparent that facilitating factors for quality measurement are to discuss quality of oral health care in a trusted environment and to develop more clear clinical practice guidelines with ready to use quality measures.

There is a willingness from all stakeholders to work on improving the quality of oral health care in a trusted environment and with a limited impact on the administrative burden. Similar to previous research in the United Kingdom, it was frequently mentioned that younger dentists were more open to quality measurement and feedback on their performance than older dentists.^19^ The reasons for willingness to invest in quality improvement do seem to differ. In the study of Byrne et al.,^19^ in the United Kingdom, the quality measures were perceived as a possible threat to the clinical autonomy and business, while in this study in the Netherlands dentists were mainly suspicious of being monitored and a possible additional workload. In both studies, concerns were raised with regard to being punished for poor outcomes. The setting and manner in which feedback is delivered can greatly impact the willingness to participate and contribute to more quality improvement.^7^^,^^20^ Feedback from quality measures can contribute to creating awareness and reflection, which may result in a change in behavior.^7^^,^^20^^,^^21^ While internationally it has been shown that routinely collecting data aiming to improve the quality of oral health care is possible, thus far this has not been the case in the Netherlands.^6^^,^^22^ A recent example from the United States shows the potential of learning and improvement towards advancing the implementation of oral health care quality measures.^23^ In the Netherlands, the fact that there is no broad consensus on what quality is and what to measure which seems to hinder next steps in quality improvement. There are other studies in which it was highlighted that a trust in the quality of the data is essential in facilitating behavior change.^24^ To be able to use quality measures for decision making measures should also be fit for purpose and actionable.^8^

A strong point of this study is that many different stakeholders were included in the study resulting in a broad overview of attitudes towards quality measurement. The findings of this study should also be interpreted considering some limitations. The findings of the study are dependent on the Dutch system and contextual conditions, meaning that the findings may not be generalisable to other countries. With the use of purposive sampling, the aim was to include participants with different views on quality of oral health care. Possible bias may have occurred since patients were approached by their dentists. Firstly, it is possible that the dentists chose patients who are more positive about the care the dentists provided. Secondly, only patients from dentists were included, people who do not see a dentist on a regular basis were not included in this study. Possibly, they have a different opinion on the quality of oral health care. Furthermore, it is possible that the participating patients had a higher socio-economic position resulting in a possible better oral health and fewer negative experiences with oral health care.^25^ Lastly, since oral health care in the Netherlands is not only provided by the dentist it is worth recommending to also include other oral health care professionals in future initiatives. However, due to the limited time and already large sample of interviews this was unfortunately not possible for this study.

This study bears practical implications to overcome barriers when measuring quality of oral health care. Next steps towards measuring quality of oral health care in the Netherlands should include discussions about what constitutes quality of oral health care and which measures can be agreed upon and implemented in practice. Previous research led to a working definition and a core set of oral health care quality measures.^9^^,^^10^ The working definition could be used as a first step to achieve consensus on a multistakeholder definition for quality of oral health care in the Netherlands. To achieve a broad consensus, it is important to not only take into account the perspectives of the various stakeholders but also understand the reason why they experience certain barriers of quality measurement. Sufficient support is necessary, and measures should be tested before use in practice. The development of an adequate automated information system that collects routinely collected feedback data based on objective observations could contribute to reducing the administrative burden and facilitate quality measurement. For the implementation of quality measurement, it is pivotal that dental students and dentists are familiar with feedback data. Discussing feedback data in a small and trusted environment could help gain support for oral health care quality measurement.

## Conclusion

This study adds to literature by describing which barriers and facilitators exist for measuring quality of oral health care in the Netherlands, which may also translate to other European countries with oral health care systems comparable to the Netherlands. It provides insights into attitudes towards oral health care quality measurement and which factors should be taken into account when aiming to improve the quality of oral health care by quality measurement. The findings signal the importance of achieving consensus on what constitutes quality of oral health care in the Netherlands and how to measure quality of oral health care. A culture shift and discussions about how quality of oral health care can be made insightful in a way acceptable to all stakeholders, are needed to advance quality improvement.

## Author contributions

Concept of design: AR, DD, KS, PW, SL; data acquisition: AR, DD, KS; data analysis: AR, DD, AO; drafting manuscript: AR; critical revision of the manuscript: AR, DD, KS, AO, PW, SL; agreement for submission and to be accountable for all aspects of the work: AR, DD, KS, AO, PW, SL.

## Conflict of interest

None disclosed.

## Appendix Supplementary materials

Image, application 1

## Footnotes

Supplementary material associated with this article can be found in the online version at doi:10.1016/j.identj.2025.03.004.
