# Supplementary material for: Stakeholders’ Perspectives on Quality Measurement of Oral Health Care in the Netherlands: A Qualitative Study
Source: Int Dent J. 2025 Apr 1;75(3):1722–31. doi: 10.1016/j.identj.2025.03.004 (PMC11999193; doi:10.1016/j.identj.2025.03.004)
Supplement: Supplementary file 1 [file mmc1.docx]

**Appendix A**

**Interview guide for patients (translated from Dutch)**

1. **Opening question**
   - This interview will be about oral health care. What comes to mind when you think of this topic?
2. **Quality of oral health care**
   - What do you understand by quality of oral health care?
   - What aspects of oral health care do you consider important?
   - What do you think is good about oral health care in the Netherlands?
   - What do you think is not so good about oral health care in the Netherlands?
3. **Measuring the quality of care**
   - We will now talk about measuring quality of care (or getting information about the quality of care). What comes to mind when I mention this?
   - Which aspects of oral health care would you like to see measured?
   - Can you indicate whether, and why, you would find it interesting to receive information about the quality of oral health care?
   - What makes measuring the quality of oral health care difficult? (barriers)
   - What could make measuring the quality of oral health care easier? (facilitators)

*The researcher provides a brief explanation of quality indicators in lay language.*

- - What do you think could make the introduction of quality indicators in dental practices challenging? (barriers to implementation)
  - Possible issues for implementation (mention specific aspects if not already discussed):
    - Related to the patient
    - Related to the dentist
    - Related to the dentist’s social environment (e.g., colleagues)
    - Related to the organization
    - Related to quality indicators
  - What do you think about measuring patient satisfaction? How do you view this?
  - If you think we need to measure this, would you prefer to answer questions via email, on paper, or using an app?

1. **Domains of quality of care**
   - If we were to divide the quality of care into different aspects, what comes to mind, and what are your thoughts on the following? (Check per interview whether the person can provide input on these items):
     - Safe oral health care
     - Effective oral health care
     - Patient-centered oral health care
     - Timely oral health care
     - Efficient oral health care
     - Equal oral health care for everyone
     - Accessible oral health care
2. **Ideal scenario**
   - Imagine there were no limitations regarding time, finances, or possibilities. What would be your ideal vision of quality of care and how it should be measured? (quality system)
3. **Dental history/additional information**
   - Finally, a few general questions:
     - What is your age?
     - Have you recently visited a dentist?
     - How many dentists/dental hygienists work at the practice where you are registered?
     - How often do you visit the dentist per year?
     - How long have you been a patient at your current practice?
     - How do you perceive the health of your own teeth?
     - Do you have additional dental insurance?
     - In which province do you live?
4. **Closing**
   - Are there any points that have not been addressed that you would like to add to this conversation?

**Interview guide for dentists (translated from Dutch)**

1. **Work as a dentist**
   - How long have you been working as a dentist?
   - Where and when did you complete your education?
   - What are your current responsibilities within the dental practice?
   - Can you describe the type of dental practice you work in?
   - How old are you?
2. **Quality of oral health care**
   - This interview will be about the quality of oral health care. What do you understand by the quality of oral health care?
   - What aspects of oral health care do you consider important?
   - What do you think is good about oral health care in the Netherlands?
   - What do you think is not so good about oral health care in the Netherlands?
3. **Domains of quality of care**
   - If we were to divide the quality of care into different aspects, what comes to mind, and what are your thoughts on the following? (Check per interview whether the person can provide input on these items):
     - Safe oral health care
     - Effective oral health care
     - Patient-centered oral health care
     - Timely oral health care
     - Efficient oral health care
     - Equal oral health care for everyone
     - Accessible oral health care
4. **Measuring the quality of care**
   - We will now talk about measuring the quality of care. What comes to mind when I mention this?
   - Which aspects of oral health care would you like to see measured?
   - Can you indicate whether, and why, you would find it interesting to receive feedback on the quality of the oral health care you provide? (Possibly in comparison to others?)
   - What makes measuring the quality of oral health care difficult? (barriers)
   - What could make measuring the quality of oral health care easier? (facilitators)

*The researcher provides a brief explanation of quality indicators in understandable language.*

- - Have you had experience with previous initiatives related to quality indicators in oral health care? If so, what has been your experience?
  - What do you think could make the introduction of quality indicators in dental practices challenging? (barriers to implementation)
  - Possible issues for implementation (mention specific aspects if not already discussed):
    - Related to the patient
    - Related to the dentist
    - Related to the dentist’s social environment (e.g., colleagues)
    - Related to the organization
    - Related to quality indicators
  - What do you think about measuring patient satisfaction? How do you view this?
  - If you were to measure this, would you prefer to answer questions via email, on paper, or using an app?

1. **Ideal scenario**
   - Imagine there were no limitations regarding time, finances, or possibilities. What would be your ideal vision of quality of care and how it should be measured? (quality system)
2. **Closing**
   - Are there any points that have not been addressed that you would like to add to this conversation?

**Interview guide for other institutions related to the quality of oral health care (translated from Dutch)**

1. **Work responsibilities**
   - What do your job responsibilities entail?
   - How are your responsibilities related to the quality of oral health care?

*Questions only to be answered by dentists:*

1. **Work as a dentist**
   - How long have you been (or were you) working as a dentist?
   - Where and when did you complete your education?
   - Can you describe the type of dental practice you work or have worked in?
2. **Quality of oral health care**
   - This interview will focus on the quality of oral health care. What do you understand by the quality of oral health care?
   - What aspects of oral health care do you consider important?
   - What do you think is good about oral health care in the Netherlands?
   - What do you think is not so good about oral health care in the Netherlands?
3. **Domains of quality of care**
   - If we were to divide the quality of care into different aspects, what comes to mind, and what are your thoughts on the following? (Check per interview whether the person can provide input on these items):
     - Safe oral health care
     - Effective oral health care
     - Patient-centered oral health care
     - Timely oral health care
     - Efficient oral health care
     - Equal oral health care for everyone
     - Accessible oral health care
4. **Measuring the Quality of Care**
   - We will now discuss measuring the quality of care. What comes to mind when I mention this?
   - Which aspects of oral health care would you like to see measured?
   - Can you indicate whether, and why, you would find it interesting to receive data on the quality of delivered oral health care? (Possibly in comparison to others?)
   - What makes measuring the quality of oral health care difficult? (barriers)
   - What could make measuring the quality of oral health care easier? (facilitators)

*The researcher provides a brief explanation of quality indicators in understandable language.*

- - Have you had experience with previous initiatives related to quality indicators in oral health care? If so, what has been your experience?
  - What do you think could make the introduction of quality indicators in dental practices challenging? (barriers to implementation)
  - Possible issues for implementation (mention specific aspects if not already discussed):
    - Related to the patient
    - Related to the dentist
    - Related to the dentist’s social environment (e.g., colleagues)
    - Related to the organization
    - Related to quality indicators
  - What do you think about measuring patient satisfaction? How do you view this?

1. **Ideal Scenario**
   - Imagine there were no limitations regarding time, finances, or possibilities. What would be your ideal vision of quality of care and how it should be measured? (quality system)
2. **Closing**
   - Are there any points that have not been addressed that you would like to add to this conversation?
